# Supplementary material for: Programming of refractive functions
Source: Nat Commun. 2025 Jul 26;16:6896. doi: 10.1038/s41467-025-62230-x (PMC12297264; doi:10.1038/s41467-025-62230-x)
Supplement: Supplementary file 1 — Supplementary Information [file 41467_2025_62230_MOESM1_ESM.pdf]

Supplementary Information for

Programming of refractive functions

Md Sadman Sakib Rahman[1,2,3]
Tianyi Gan[1,3]
Mona Jarrahi[1,3]
Aydogan Ozcan[1,2,3,*]

[1]Electrical and Computer Engineering Department, University of California, Los Angeles, CA, 90095, USA
[2]Bioengineering Department, University of California, Los Angeles, CA, 90095, USA
[3]California NanoSystems Institute (CNSI), University of California, Los Angeles, CA, 90095, USA
[*]Corresponding author: ozcan@ucla.edu

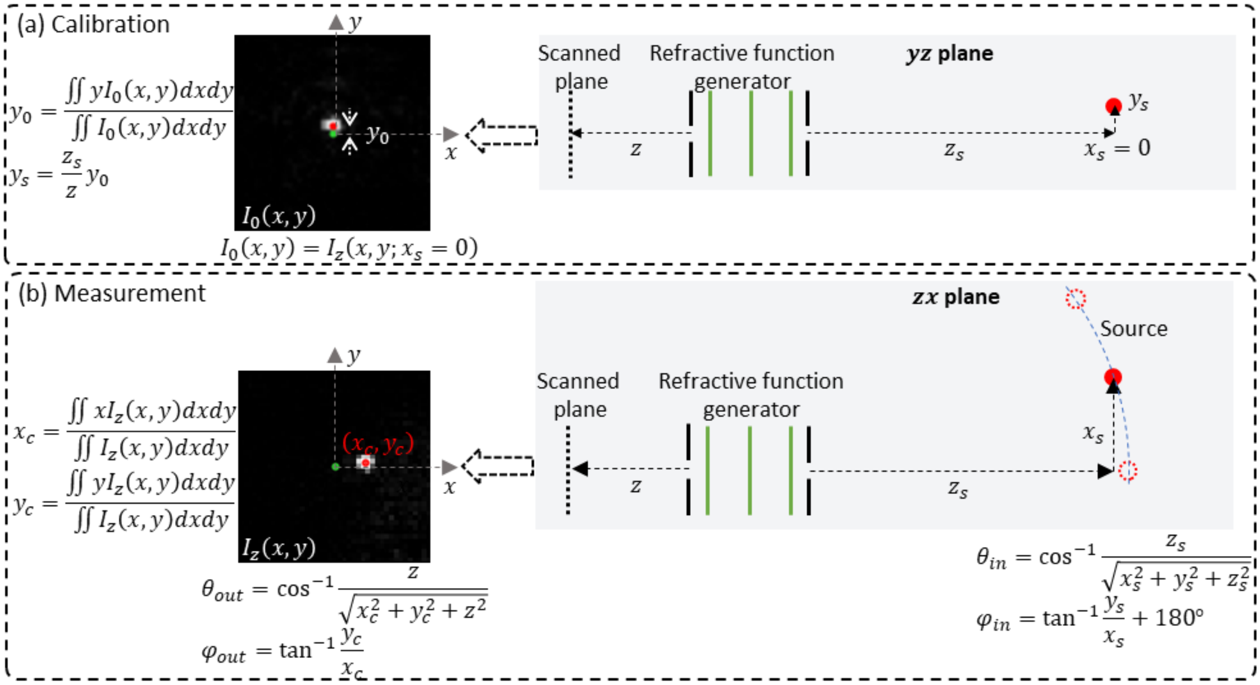

Fig. S1: Schematics outlining the experimental measurement procedure. (a) Calibration step to correct the height y_s of the source relative to the input aperture. y_s was calculated from the y-coordinate (y_0) of the first moment of the diffracted pattern $I_0(x,y)$ when the x-displacement of the source relative to the input aperture $x_s = 0$. (b) To obtain measurements corresponding to different input directions, the source was moved horizontally (constant y_s) along an arc to vary θ_{in}; for a given θ_{in}, the variation of φ_{in} was realized by rotating the RFG surfaces in plane relative to the static source. For each measurement, the x_s and z_s of the source relative to the input aperture were recorded. x_s, y_s and z_s were used to

estimate θ_{in} and φ_{in}, and the first moment (x_c, y_c) of the diffracted intensity pattern at a distance $z = 80$ mm from the output aperture was used to estimate θ_{out} and φ_{out}.

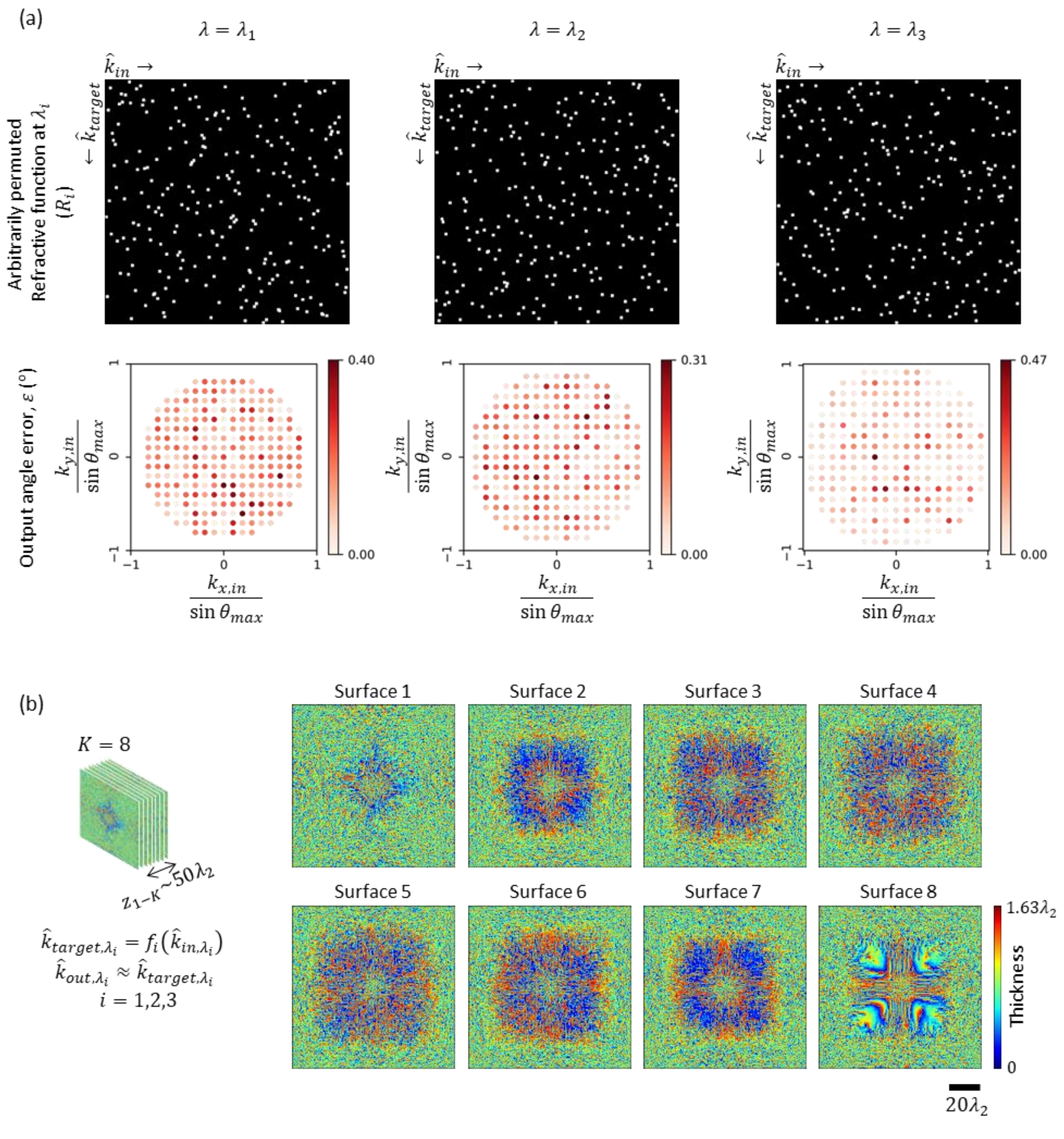

Fig. S2: Wavelength multiplexing of arbitrarily permuted refractive functions with an RFG design comprising a material with no dispersion, i.e., $n_1=n_2=n_3=n$. (a) The matrices representing the targeted arbitrarily permuted refractive functions at three distinct wavelengths (top row). The bottom row shows, for a $K = 8$ RFG design, the error in the output angle as a function of the input direction at these three wavelengths. (b) The optimized thickness profiles of the RFG surfaces. The distance z_{ll} between consecutive surfaces is $\sim 6\lambda_2$, giving an axial distance of $z_{1-K} \approx 50\lambda_2$ between the first and the last transmissive surfaces.

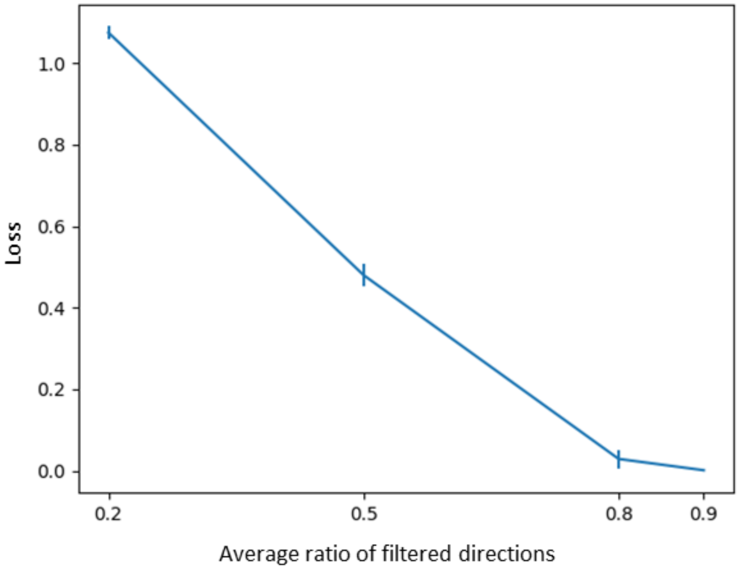

Fig. S3: Training loss as a function of the ratio of filtered directions for implementing arbitrarily filtered and permuted refractive functions. The loss is calculated by using Eq. 14 in the main text and averaging over all the input directions. The error bar arises from three different designs with the same average ratio of filtered directions but with differently selected filtered directions and refractive permutation functions.

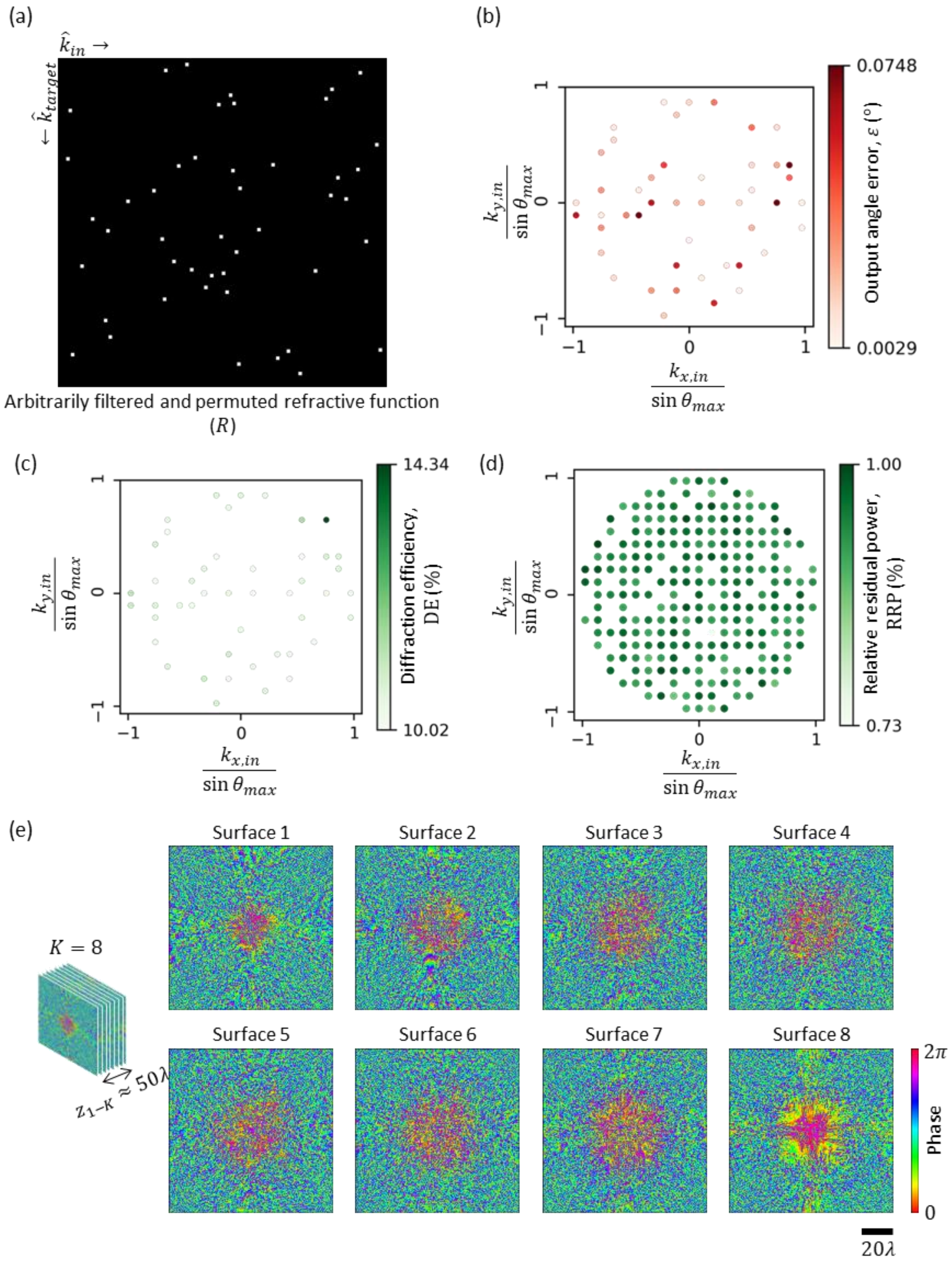

Fig. S4: Arbitrarily filtered and permuted refractive function implementation with a $K = 8$ RFG design. (a) The matrix R representing an arbitrarily filtered and permuted refractive function, which is the same as the one depicted in Fig. 1c (column 4) of the main text. The ratio of the filtered directions is ~80%. (b) Output angle error ε for all the unfiltered input directions. (c) Output diffraction efficiency DE for all the

unfiltered input directions. (d) Relative residual power RRP (see Eq. (16) in the main text) for the filtered input directions. (e) The optimized phase profiles of the RFG surfaces.

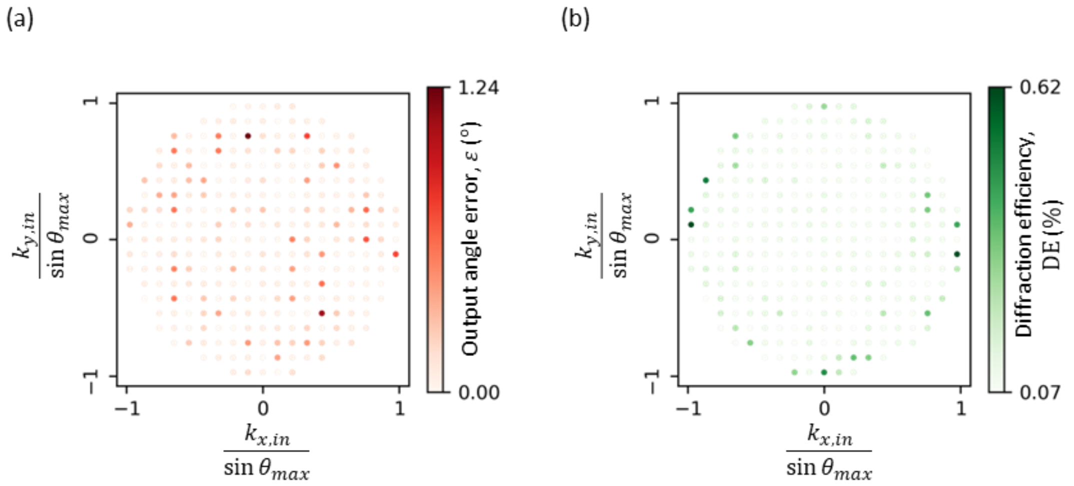

Fig. S5: Performance of an RFG, designed for a target refractive function f in the forward (axial) direction, when it is blindly tested for propagation in the reverse (axial) direction. Here, the refractive function f is the same as the one reported in Fig. 2 of the main text. For this evaluation, however, the target directions in the reverse path are set by f^{-1}. (a) Output angle errors for propagation in the reverse direction. (b) Diffraction efficiencies for propagation in the reverse direction.

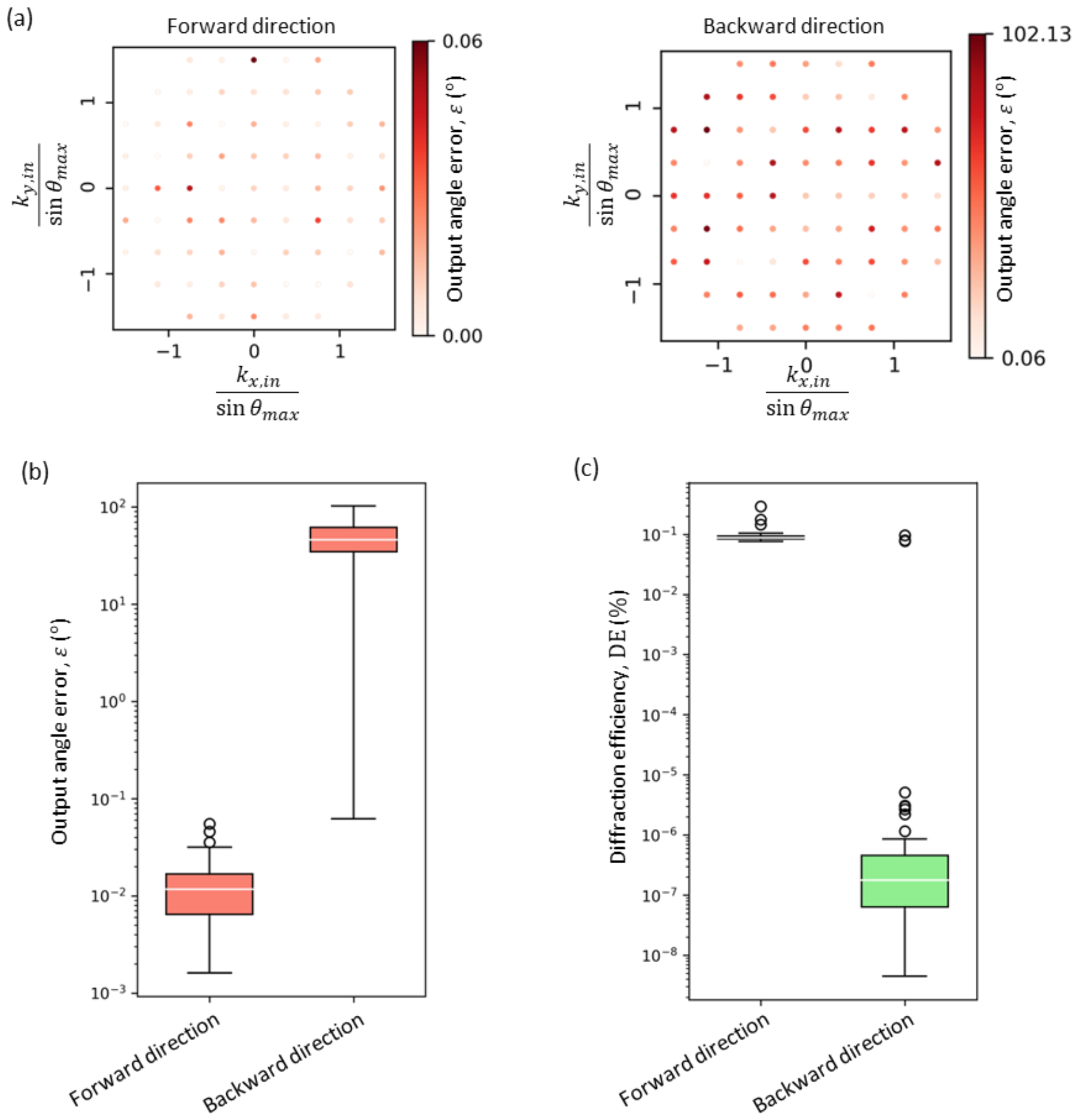

Fig. S6: Unidirectional RFG design for an arbitrary permutation refractive function, optimized by only suppressing the backward transmission. (a) Output angle error across the grid of directions for forward propagation (left) and backward propagation (right). (b) Distribution of angular errors in both directions. (c) Distribution of diffraction efficiencies in both directions, showing effective suppression of the backward transmission (six orders of magnitude difference in the median diffraction efficiencies along the forward and backward directions).

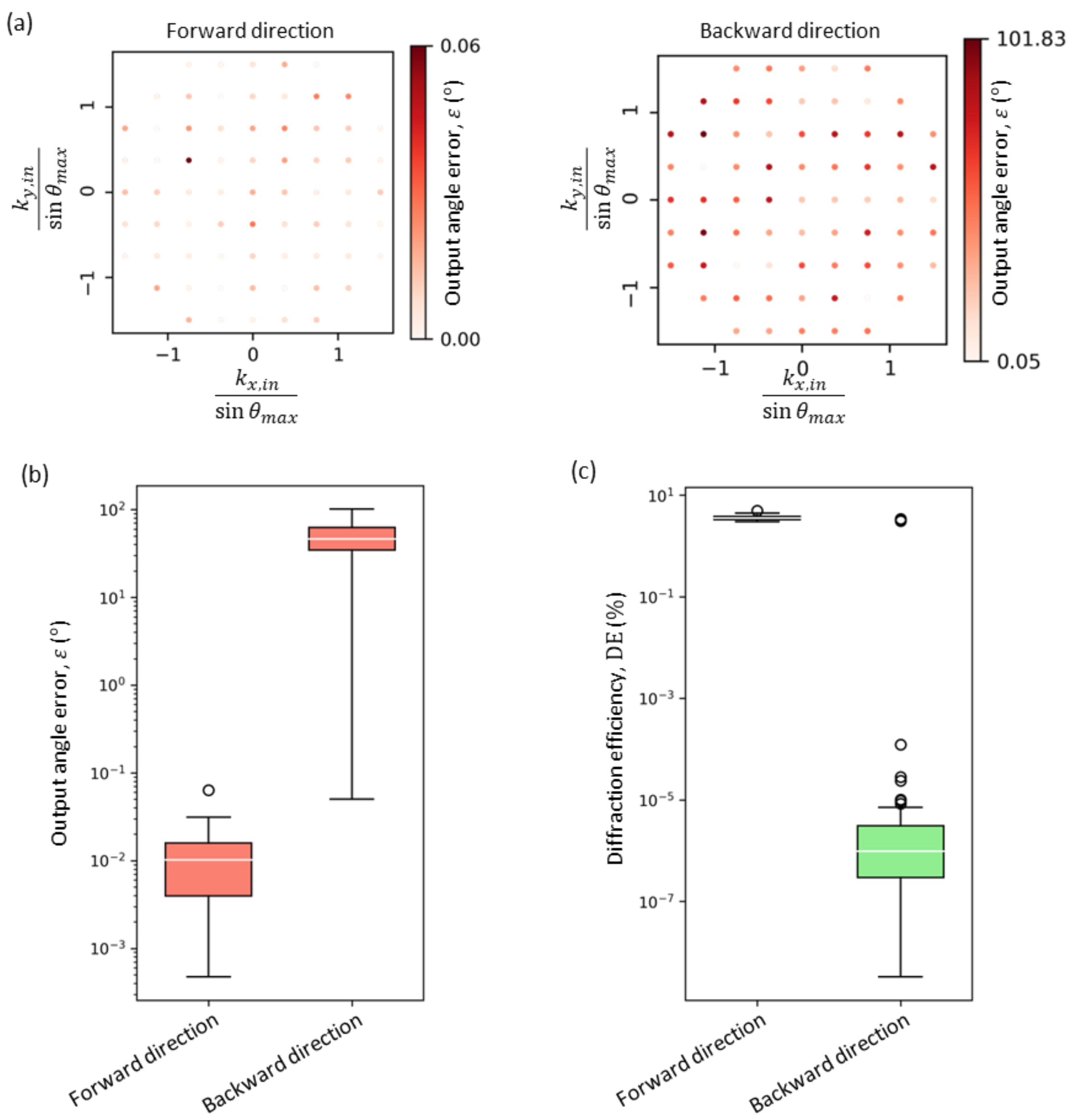

Fig. S7: Unidirectional RFG design for an arbitrary permutation refractive function, optimized by both boosting the forward transmission and suppressing the backward transmission. (a) Output angle error across the grid of directions for forward propagation (left) and backward propagation (right). (b) Distribution of angular errors in both directions. (c) Distribution of diffraction efficiencies in both directions, showing effective suppression of the backward transmission (more than six orders of magnitude difference in the median diffraction efficiencies along the forward and backward directions).

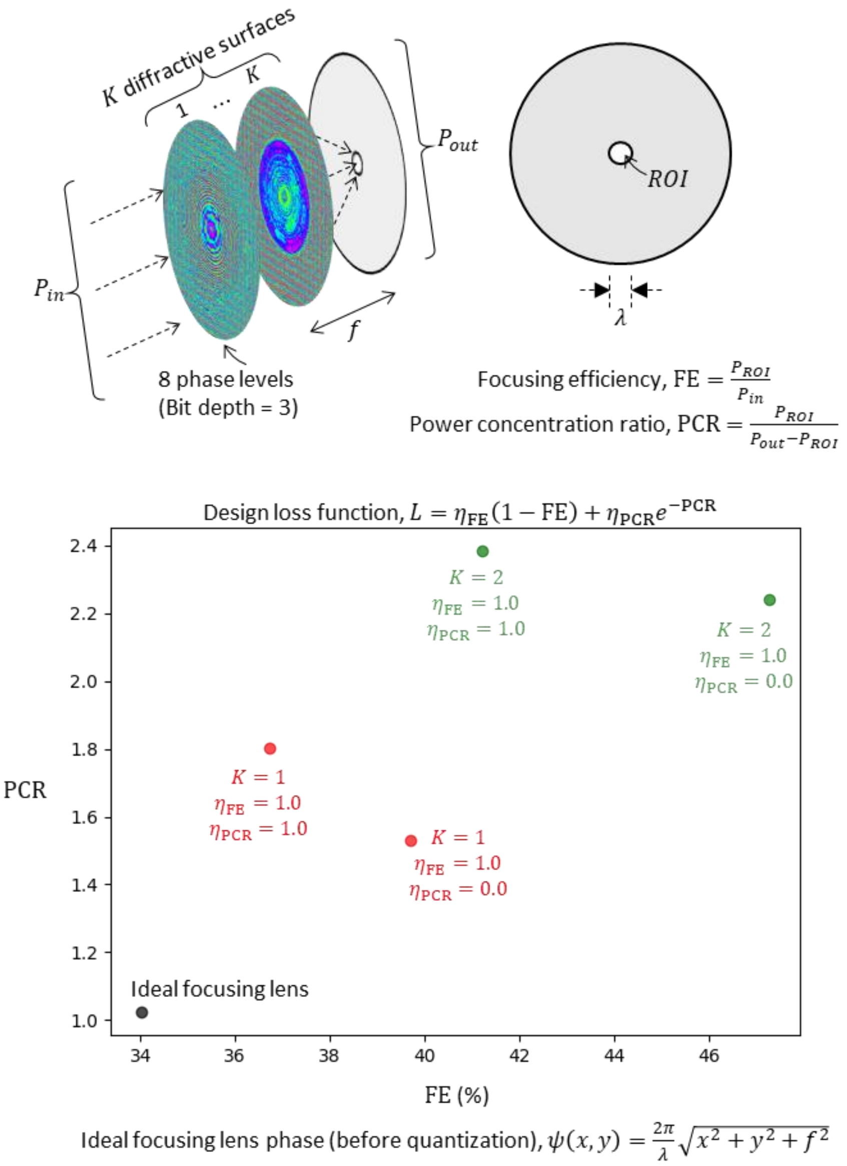

Fig. S8: Diffractive focusing optics using physics-based learning of cascaded surfaces. Top left: A schematic showing K transmissive surfaces, composed of 8-level-quantized phase-elements (i.e., 3 phase bit-depth), for focusing light within a spot of diameter λ at a focal length of f. Top right: Illustration of the region of interest (ROI) at the output plane, i.e., the focal spot of diameter λ, used to compute the focusing efficiency (FE) and power concentration ratio (PCR). Bottom: Performance comparison of learned diffractive focusing with $K = 1$ and $K = 2$, and an ideal focusing lens phase profile, under the same assumption of spatial discretization and 3 bit-depth phase quantization. The learned designs demonstrate higher performance in both metrics and allow controlled trade-offs between these metrics through loss function engineering.

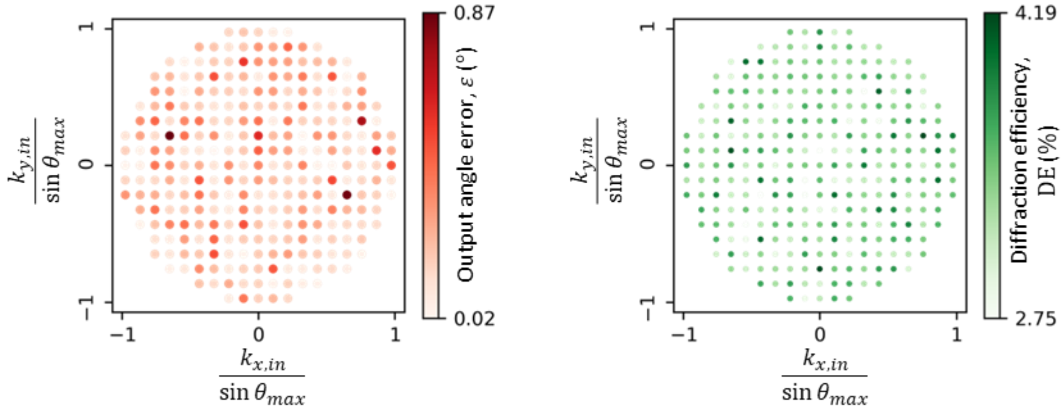

Fig. S9: Performance of an RFG design implemented in the visible spectrum using a low-loss material. Angular error and diffraction efficiency of the RFG designed with $\eta = 30$ in Fig. 4 of the main text, evaluated at 562.5 nm illumination wavelength. The same optimized phase profiles are used, while the physical dimensions are scaled proportionally to the new operation wavelength. The diffractive surfaces are assumed to be fabricated using PMMA, with a refractive index of 1.4863 and an extinction coefficient of 2.27×10^{-7} at 562.5 nm. Compared with the terahertz design assuming no absorption (see Fig. 4 in the main text), no perceptible change in performance is observed, confirming that the assumption of negligible absorption is valid.

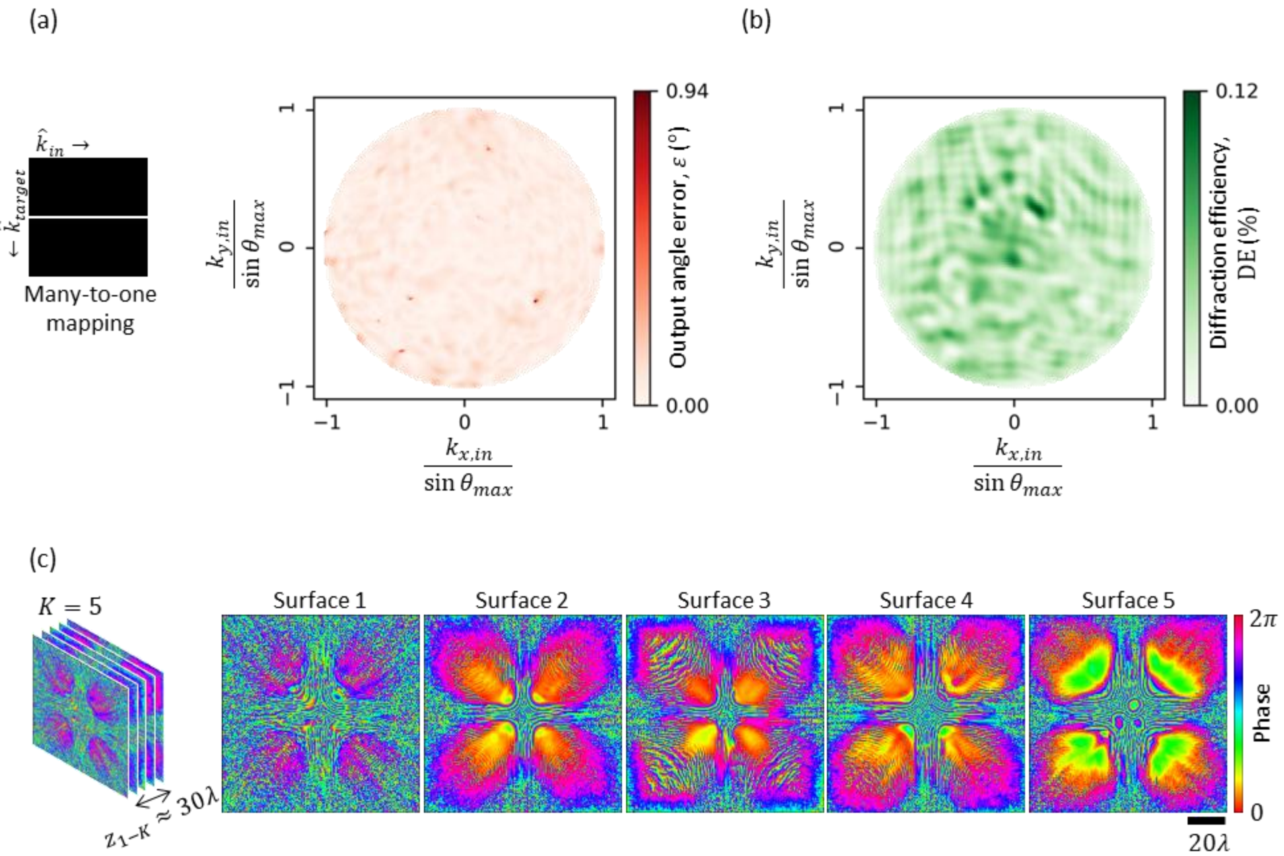

Fig. S10: Many-to-one refractive function realization using an RFG. (a) The RFG is trained to implement a many-to-one angular transformation, where all the input directions of interest are mapped to the same output direction. Output angle error for all the input directions, sampled densely within θ_{max}, remain below 1°. (b) Diffraction efficiency for all the input directions. (c) The optimized phase profiles of the RFG surfaces. The axial distance z_{ll} between consecutive surfaces is $\sim 6\lambda$, giving an axial distance of $z_{1-K} \approx 30\lambda$ between the first and the last transmissive surfaces.

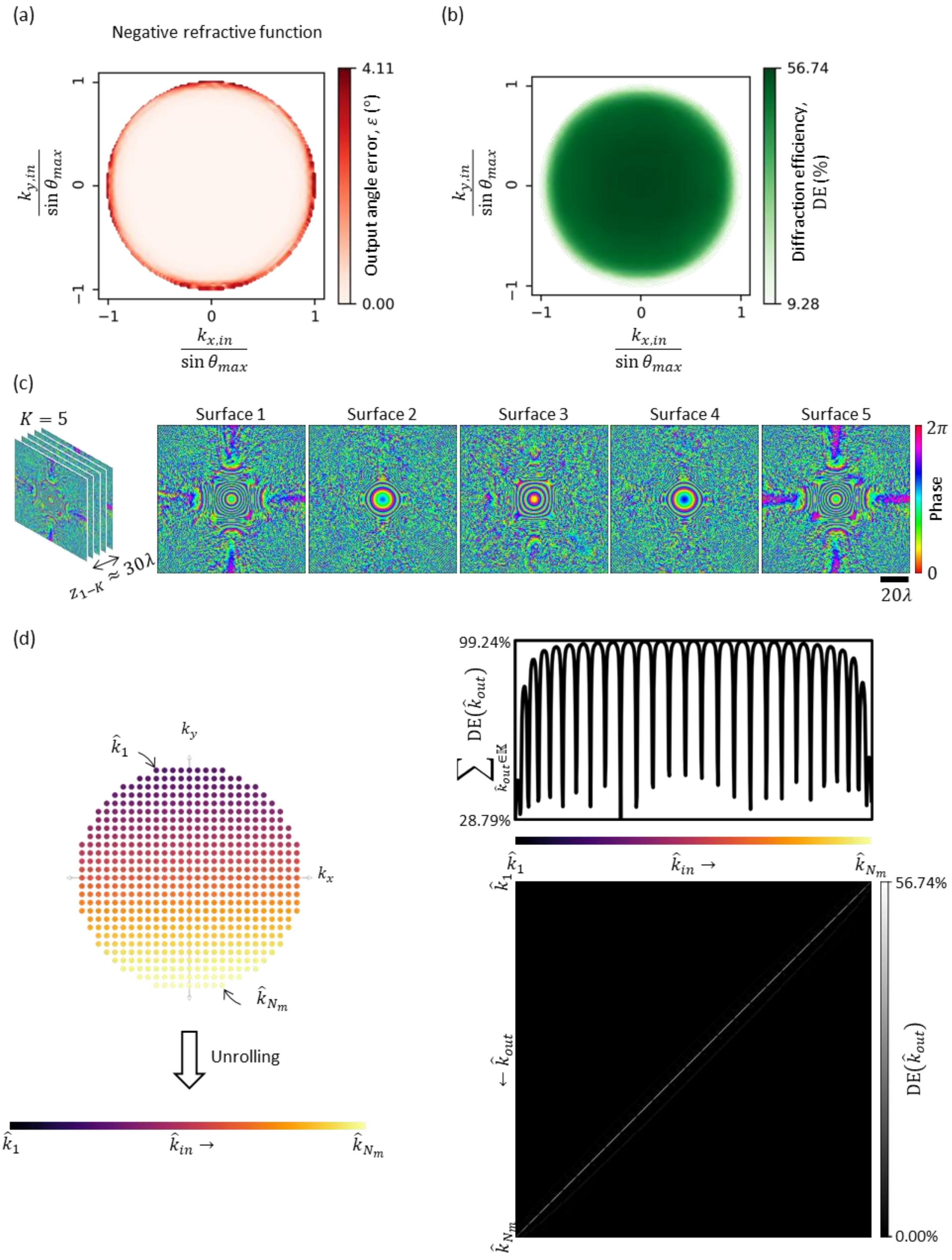

Fig. S11: Efficiency-enhanced RFG design for negative refractive function. (a) Output angle error for all

the input directions, sampled densely within θ_{max}. (b) Diffraction efficiency for all the input directions. (c) The optimized phase profiles of the RFG surfaces. (d) Analysis of optical power distribution. The space of input (and output) directions is discretized with an interval of $\frac{\lambda}{D_a}$ and subsequently linearized into a one-dimensional vector. The diffraction efficiencies along all the output directions $\hat{k}_{out}$ for a given input direction $\hat{k}_{in}$ are shown via the 'confusion matrix' on the bottom right. The dominant diagonal structure reveals that most of the output power is correctly directed into the intended target angles defined by the negative refractive function, while residual off-target leakage is observed—particularly when θ_{in} is close to θ_{max}. The total diffraction efficiency over all the output directions can approach 99.24%, as shown on the top-right plot obtained by summing over the rows of the 'confusion matrix'. The observed dips in diffraction efficiency correspond to $\theta_{in} \approx \theta_{max}$.

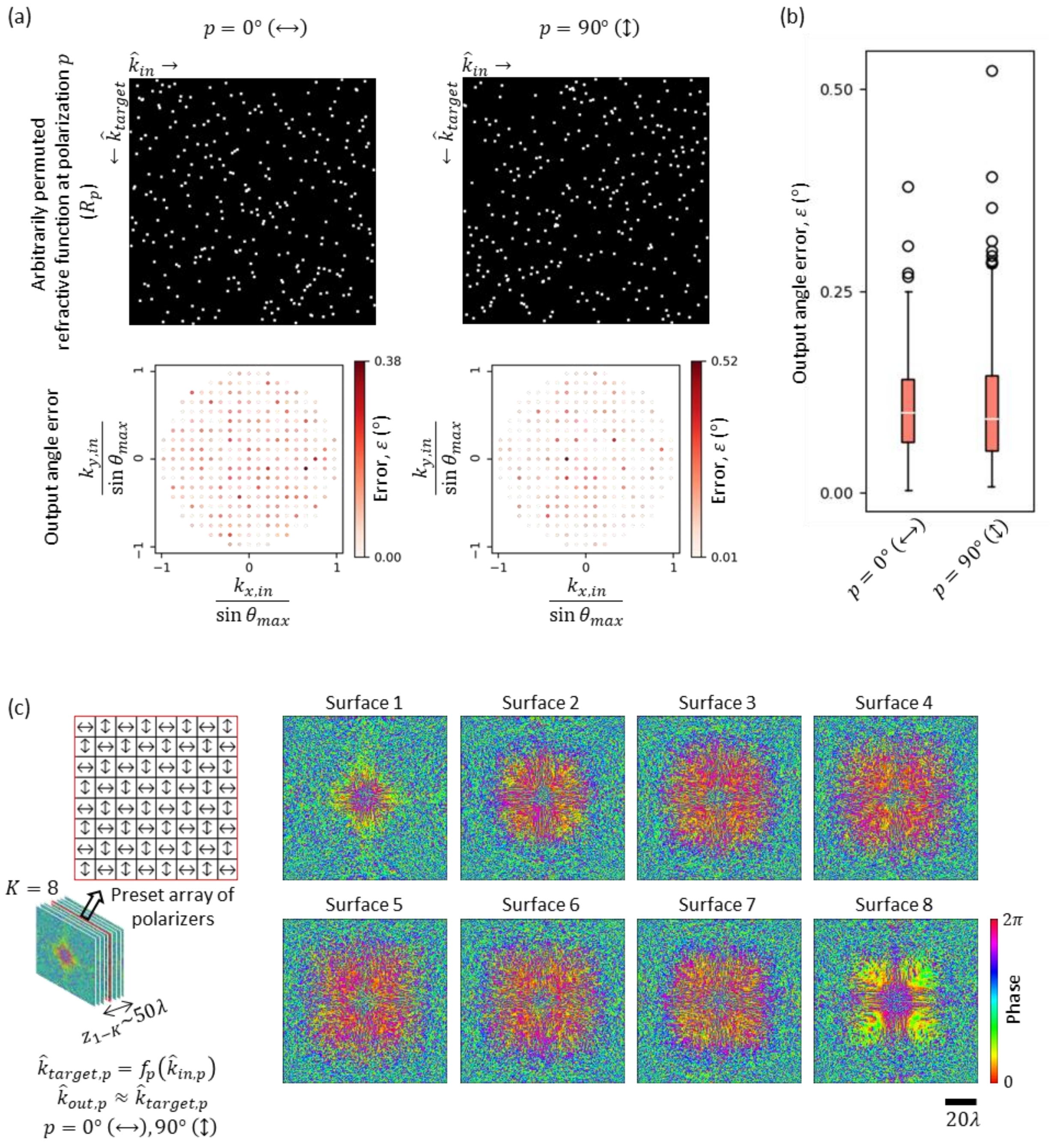

Fig. S12: Polarization multiplexing of two arbitrary permutation refractive functions with an RFG by using a preset/predetermined array of polarizers. (a) The matrices representing the targeted arbitrary permutation refractive functions for two orthogonal linear polarization states: horizontal ($p = 0°$, left) and vertical ($p = 90°$, right). The bottom row shows the output angle error as a function of the input direction for each polarization state. (b) Distribution of angular error over all input directions for the two polarization states. (c) A schematic showing the placement of the polarizer array within the RFG, together with the optimized phase profiles of the RFG surfaces. The arrows representing the polarizers denote the orientation of the transmission axis. The width of each polarizer on the 8×8 array is assumed to be $\sim 13.3\lambda$.

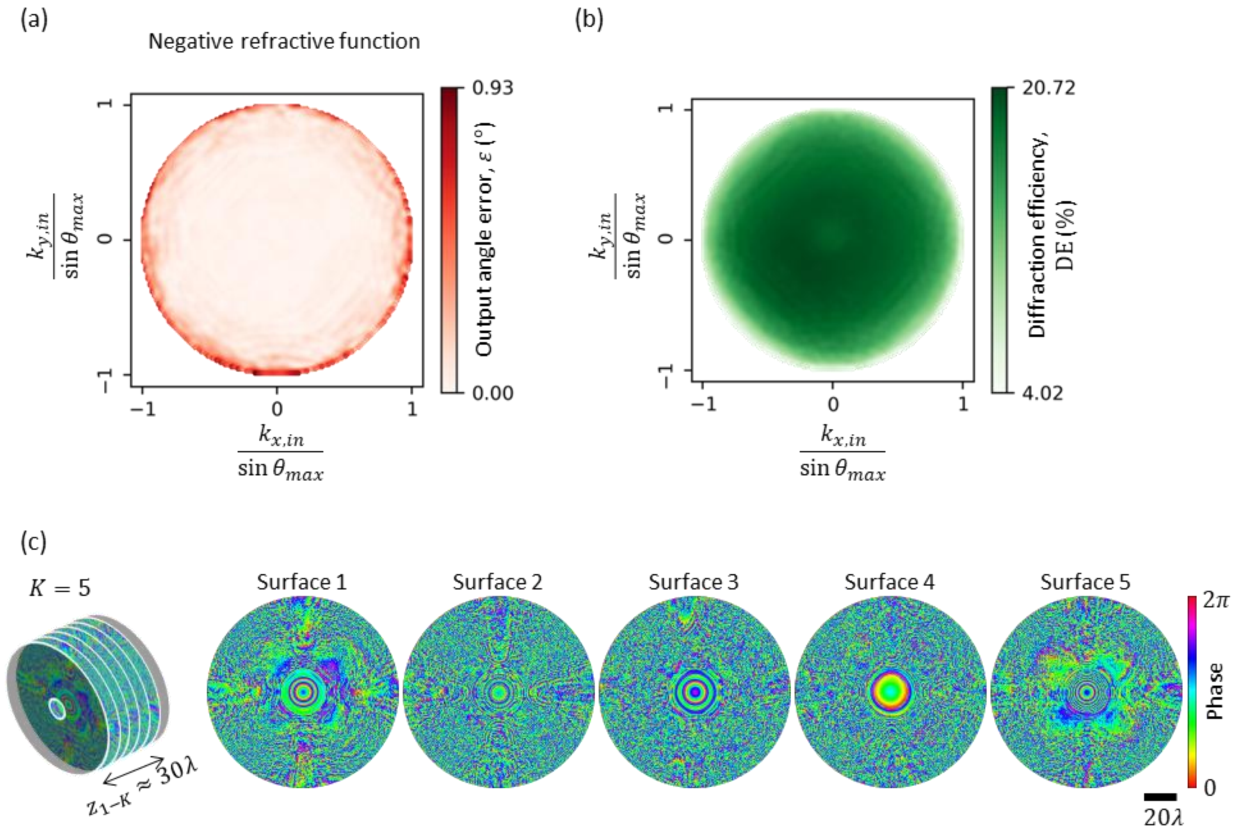

Fig. S13: Negative refractive function RFG with a circular aperture. (a) Output angle error for all the input directions, sampled densely within θ_{max}. (b) Diffraction efficiency for all the input directions. (c) The optimized phase profiles of the RFG surfaces.
