# Supplementary material for: Programming of refractive functions
Source: Nat Commun. 2025 Jul 26;16:6896. doi: 10.1038/s41467-025-62230-x (PMC12297264; doi:10.1038/s41467-025-62230-x)
Supplement: Supplementary file 2 — Description of Additional Supplementary Files [file 41467_2025_62230_MOESM2_ESM.pdf]

Description of Additional Supplementary Files

Supplementary Movie 1: Far-field intensity patterns of an arbitrary permutation refractive function generator for different input directions. For the RFG described in Fig. 2 of the main text, the far-field intensity patterns are shown as the input wave directions are swept over. The intensity patterns are calculated at a plane that is 160λ away from the output aperture. For comparison, the far-field patterns corresponding to the target wavefronts are also shown.

Supplementary Movie 2: Far-field intensity patterns corresponding to wavelength multiplexing of arbitrary permutation refractive functions. For the wavelength-multiplexed RFG described in Supplementary Fig. S2, the far-field intensity patterns are shown as the input wave directions are swept over at the design wavelengths. The far-field patterns corresponding to the target wavefronts are also shown for comparison.

Supplementary Movie 3: Far-field intensity patterns of a many-to-one refractive function generator for different input directions. For the RFG described in Supplementary Fig. S10, the far-field intensity patterns are shown as the input wave directions are swept over. The intensity patterns are calculated at a plane that is 160λ away from the output aperture. For comparison, the far-field patterns corresponding to the target wavefronts are also shown.

Supplementary Movie 4: Far-field intensity patterns corresponding to polarization multiplexing of two arbitrary permutation refractive functions. For the polarization-multiplexed RFG described in Supplementary Fig. S12, the far-field intensity patterns are shown as the input wave directions are swept over at the corresponding polarization states. The far-field patterns corresponding to the target wavefronts are also shown for comparison.
